# Evaluating SARS-CoV-2 T Cell Immunity in COVID-19-Naive Vaccinated Individuals with and Without Spike Protein IgG Antibodies

**DOI:** 10.3390/pathogens14050415

**Published:** 2025-04-25

**Authors:** Vassiliki C. Pitiriga, Myrto Papamentzelopoulou, Dimitris Nikoloudis, Chrysa Saldari, Kanella E. Konstantinakou, Irene V. Vasileiou, Athanasios Tsakris

**Affiliations:** 1 Department of Microbiology, Medical School, National and Kapodistrian University of Athens, 75 Mikras Asias Street, 11527 Athens, Greece; chrysasaldari@gmail.com (C.S.); atsakris@gmail.com (A.T.); 2 Molecular Biology Unit, 1st Department of Obstetrics and Gynecology, National and Kapodistrian University of Athens, 11527 Athens, Greece; mpntua@yahoo.gr; 3 Bioiatriki Healthcare Group, Kifisias 132 and Papada Street, 11526 Athens, Greece; dnicolgr@hotmail.com (D.N.); nkonstantinakou@bioiatriki.gr (K.E.K.); irenebasileiou@gmail.com (I.V.V.)

**Keywords:** COVID-19, SARS-CoV-2, vaccination, T-SPOT, cellular immunity, T cell

## Abstract

**Background:** The effective management of vaccination schedules requires thorough knowledge of an individual’s immunoprotection level, including the interaction and persistence of immune responses at both the humoral and cellular levels following SARS-CoV-2 vaccination. This study aimed to investigate the potential relationship between the levels and duration of the SARS-CoV-2 T cell response and IgG measurements in a cohort of COVID-19-naive individuals who had received the SARS-CoV-2 vaccine. **Methods:** We performed a retrospective descriptive analysis utilizing data retrieved from the electronic medical records of consecutive COVID-19-naive and vaccinated adult individuals who underwent COVID-19 immunity screening at a private healthcare center from September 2021 to September 2022. T cell response was evaluated using the IGRA methodology T-SPOT^®^.COVID (Oxford Immunotec, Abingdon, Oxfordshire, UK). **Results: **A retrospective analysis was conducted on a cohort of 262 individuals, comprising 148 females (56.5%) and 114 males (43.5%), with ages ranging from 17 to 92 years (mean age: 59.47 ± 15.5 years). Among the participants, 216/262 (82.4%) tested negative for SARS-CoV-2 IgG antibodies (group A), while 46/262 (17.6%) tested positive (group B). No significant difference was observed between the two groups in the time period post vaccination, with the mean times after vaccination being 136.38 ± 78.68 days in group A and 140.6 ± 79.5 days in group B (T-test, *p* = 0.74). Among the two groups, a positive T cell reaction against the S antigen was reported in 132/216 (61.1%) participants in group A and 39/46 (84.8%) in group B (*X*^2^ test, *p* = 0.002). Additionally, individuals with a positive antibody response demonstrated statistically significant higher T SPOT results compared to those with undetectable antibody levels, with a mean SFC count of 125.70 for group A and 158.73 for group B (Mann–Whitney test, *p* = 0.006). **Conclusions:** Our findings suggest that T cell immunity may persist even when antibodies are undetectable, highlighting the potential role of cellular immunity in providing protection against COVID-19 over time. Additionally, this study demonstrates a significant correlation between humoral and cellular immune response levels to SARS-CoV-2, suggesting that the activation of humoral immunity following SARS-CoV-2 vaccination is associated with higher levels of cellular immunity compared to individuals with undetectable antibody levels.

## 1. Introduction

SARS-CoV-2 vaccination has been recognized as an effective means of reducing the risk of both infection and reinfection, as well as preventing severe disease, hospitalization and death [1]. Protection against COVID-19 infection is measured mostly by the level of antibodies produced against SARS-CoV-2 and their neutralizing capacity. Nevertheless, the significance of T cell responses in fighting COVID-19 infection is highlighted in several studies, demonstrating the longevity and persistence of cellular immunity regardless of the co-existence of humoral responses [2,3,4,5,6,7,8]. Vaccine-mediated immune reactions have been proven to reach their maximum values approximately 14 days after a complete double vaccination regimen [9].

Regarding both vaccine-induced cellular and humoral immunity, sufficient anti-spike (anti-S) IgG antibodies and T cell responses were presented in vaccinated SARS-CoV-2-naive individuals up to 3 months after vaccination [10]. Furthermore, mRNA vaccination induced antigen-specific CD4+ and CD8+ T cells, and early CD4+ T cell responses were correlated with long-term humoral immunity [11]. In another study, the level of neutralizing activity remained higher than 75% almost 6 months post complete vaccination, despite the mild waning of the SARS-CoV-2 anti-S IgG antibodies [12]. In a longitudinal analysis, SARS-CoV-2-naive vaccinees exhibited coordinated humoral and cellular reactions with distinct trajectories post complete mRNA vaccination [13]. A study involving mRNA-vaccinated healthcare professionals showed that antibody titers fell to a median of 178.02 binding antibody units (BAU)/mL within 7–9 months after vaccination, presenting a moderate positive correlation with the vaccination-induced cellular responses [14].

However, longitudinal studies are showing that the level of immunization diminishes with time, particularly in terms of antibody levels. As demonstrated, robust and stable T cell immunological reactions were detected up to 6 months post complete vaccination with substantial declines in humoral responses [15]. In another study, the levels of anti-S IgG antibodies waned significantly 3 months after the second dose, while T cell responses were maintained at 86% [16]. In a study of mRNA-vaccinated SARS-CoV-2-naive individuals, a decline of up to 38% each subsequent month from the highest mean antibody levels was reported, with 16.1% of study participants presenting antibody levels below the seropositivity threshold 6 months post vaccination [17]. Moreover, anti-RBD IgG titers waned significantly between 3 and 6 months post vaccination in uninfected COVID-19 healthcare workers, declining by approximately 50–65%. On the other hand, spike-specific T cell responses presented a milder decrease of approximately 18–40% between 3 and 6 months after the second dose, depending on the vaccination regimen [18]. In a follow-up analysis, neutralizing activity against SARS-CoV-2 waned 4 months after complete vaccination, while T cell immunological reactions were more stable and conserved up to 5 months, irrespective of the vaccination regimen [19]. Moreover, in specific populations with autoimmune diseases, the administration of immune-modulating therapies may reduce the SARS-CoV-2-specific immune response following COVID-19 vaccination [20,21].

In our previous survey [22], we demonstrated that cellular immune responses remained stable, despite declining antibody levels over time, in a COVID-19-naive vaccinated population. To further explore the complexity of interactions between cellular and humoral SARS-CoV-2 immunity, in the present cross-sectional study, we divided the population of our cohort into two groups: those who maintained their antibody levels after vaccination and those who did not. We aimed to examine and compare the following: (a) the development or absence of cellular immunity, (b) the quantitative levels of cellular immunity and (c) the temporal correlation between antibody levels and cellular immunity. Given the variability in the humoral and cellular immune responses induced by SARS-CoV-2 vaccination, particularly in terms of both breadth and duration, this study will contribute to a better understanding of the longevity and dynamics of adaptive immune responses in healthy vaccinees.

## 2. Materials and Methods

### 2.1. Study Design

We performed a retrospective descriptive analysis of data collected from the electronic medical records of adult individuals who had proceeded consecutively to ‘Bioitriki’ Healthcare center, a large medical center in the region of Attica, from September 2021 to September 2022, in order to be examined on their own initiative for SARS-CoV-2 immunity screening during the pandemic.

COVID-19-naive (non-exposed) and previously fully vaccinated individuals were included in this study. Requirements for enrollment included no current or prior signs or symptoms of COVID-19, no known contact with a confirmed SARS-CoV-2-infected individual and no diagnosis with SARS-CoV-2 infection prior to vaccination, based on PCR results, rapid tests, self-tests and specific IgG IgM antibody measurements. We included subjects who had completed their primary vaccination series and had not received any booster doses until the examination day. Immunocompromised patients were excluded from this study. The date of the initial vaccination completion (date of 1st or 2nd dose administration, depending on the type of vaccine) was considered as the date of vaccination. Additional data in terms of medical history were obtained by using structured questionnaires that were routinely filled by all individuals at the time of examination. No precise information was obtained for all individuals regarding the exact type of vaccines administered; however, mRNA vaccines were largely available to the majority of Greek population during that period. This study was approved by the institutional review board (Date of approval: 29 June 2021, 6th annual Meeting).

### 2.2. Laboratory Methods

#### 2.2.1. Enzyme-Linked Immunosorbent Spot (ELISpot) Assay for IFN-g T Cell Response Detection

The T cell response to SARS-CoV-2 infection was estimated by performing the IGRA methodology T-SPOT^®^.COVID (Oxford Immunotec, Abingdon, Oxfordshire, UK). The T-SPOT.*COVID* test is a standardized ELISpot-based technique for the detection of the T cell immune response to SARS-CoV-2 in whole blood. The test uses the established T-SPOT Technology with an antigen mix, based on SARS-CoV-2 structural proteins, spike (S) and nucleocapsid (N).

Blood samples were drawn into lithium heparin tubes where the T cell *xtend* reagent (Oxford Immunotec, Abingdon, Oxfordshire, UK) was added. Peripheral blood mononuclear cells (PBMCs) were then separated from a whole blood sample, washed and then counted before being added into the test. An estimated number of 250,000 cells/well were plated into 4 wells of a 96-well plate.

The two antigen peptide pools were added to the two antigen wells; the T cell mitogen phytohemagglutinin was added to the positive control well and cell culture media alone to the negative control well. The wells were washed after a period of 16–20 h of incubation, and a conjugated secondary antibody was added with the capacity to bound to any IFN-γ captured on the membrane. In this process, the wells were washed to remove the unbound IFN-γ, and a substrate was added to produce the characteristic dark spots of insoluble products indicating areas of IFN-γ presence. Spot-forming cells (SFCs)/250,000 PBMCs were manually counted using microscopy by expert and experienced technologists. The results were reported separately for the N and S antigens. They were expressed as ‘invalid’ if the negative control had more than 10 SFCs or the positive control had fewer than 20 SFCs when the antigen wells were non-reactive. The test cut-off was predefined at 6 SFCs for each antigen. A borderline zone of ±1 SFCs was introduced to account for potentially elevated test variability around the cut-off. Accordingly, the results were reported as S- and/or N-reactive when the number of SFCs in the S or/and N antigen well minus the negative control was ≥8; S- and N-non-reactive when the number of SFCs in the respective antigen wells minus the negative control was ≤4; and S- or/and N-borderline when the number of SFCs in the respective antigen well minus the negative control was 5, 6 or 7.

#### 2.2.2. SARS-CoV-2 IgG Antibodies

SARS-CoV-2 IgG antibody levels were measured in participants’ blood sera the same day of examination, using the SARS-CoV-2 IgG II Quant assay (Abbott Laboratories, Abbott Park, IL, USA) (quantitative method). This is an automated, two-step, chemiluminescent microparticle immunoassay (CMIA). It is used for the qualitative and quantitative determination of IgG antibodies to the receptor binding domain (RBD) of the S1 subunit of the spike protein of SARS-CoV-2 in human serum and plasma on the Alinity i system (Abbott Laboratories, IL, USA). The sequence used for the RBD was taken from the WH-Human 1 coronavirus, GenBank accession number MN908947. The analytical measurement interval is stated by the manufacturer as 21–40,000 arbitrary units (AU)/mL, and the reported positivity cut-off is ≥50 AU/mL.

### 2.3. Statistical Analysis

The chi-square (*Χ*^2^) test was performed to evaluate differences between categorical variables and a one-way ANOVA or T-test to compare continuous variables. In cases of non-parametric continuous variables, a Mann–Whitney test was carried out. Spearman’s rank test was performed to examine possible correlations between T SPOT SFC counts, IgG antibodies and the time interval post vaccination.

Logistic regression analysis was performed to assess the association between age, days since vaccination, IgG antibody titers, comorbidities and sex with SARS-CoV-2-specific response levels. Spearman’s rank correlation was used to assess associations between continuous variables, while point biserial correlation was used for relationships between continuous and categorical variables. Correlation coefficients (ρ for Spearman’s rank and r for point biserial) and the corresponding *p*-values were reported. The kinetics of the SFC counts of the S antigen over time were exhibited through the plotting charts of the curve estimation for linear regression models. A significance threshold of *p* < 0.05 was applied for all statistical tests. To control for potential Type I error due to multiple comparisons, Bonferroni correction was applied where appropriate. The statistical analysis and graphical representations were carried out using SPSS version 28 (IBM SPSS Statistics).

## 3. Results

### 3.1. Participants’ Demographic Characteristics

**Figure 1:**
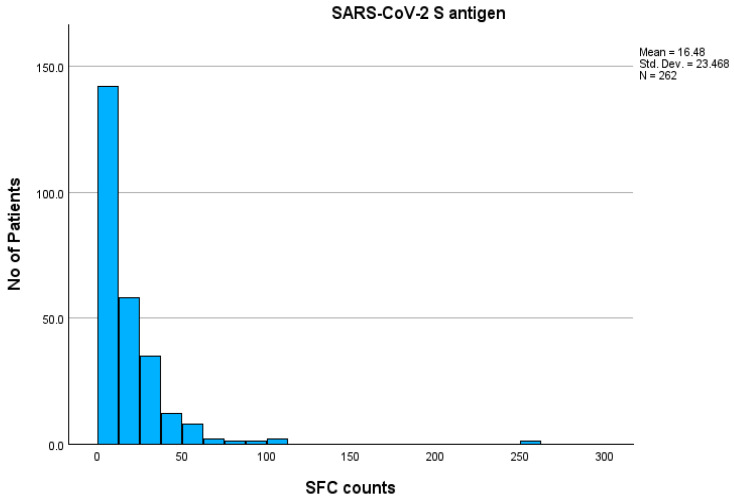
Distribution of T-SPOT values against S antigen among total participants. **Abbreviations:** S antigen—Spike protein antigen (of SARS-CoV-2); SARS-CoV-2—Severe Acute Respiratory Syndrome Coronavirus 2; T-SPOT—T cell Spot Test.

A cohort of 262 individuals, comprising 148 females (56.5%) and 114 males (43.5%), with ages ranging from 17 to 92 years (mean age: 59.47 ± 15.5 years), were included in this study. The mean T-SPOT SFC value was 16.48 (±23.47). Among the participants, 216/262 (82.4%) tested negative, while 46/262 (17.6%) tested positive for SARS-CoV-2 IgG antibodies (group A and group B, respectively). Additionally, 91/262 (34.7%) exhibited a negative T-SPOT response, whereas 171/262 (65.3%) had a positive T-SPOT response. The distribution of T-SPOT values among total participants is presented in Figure 1.

**Figure 2:**
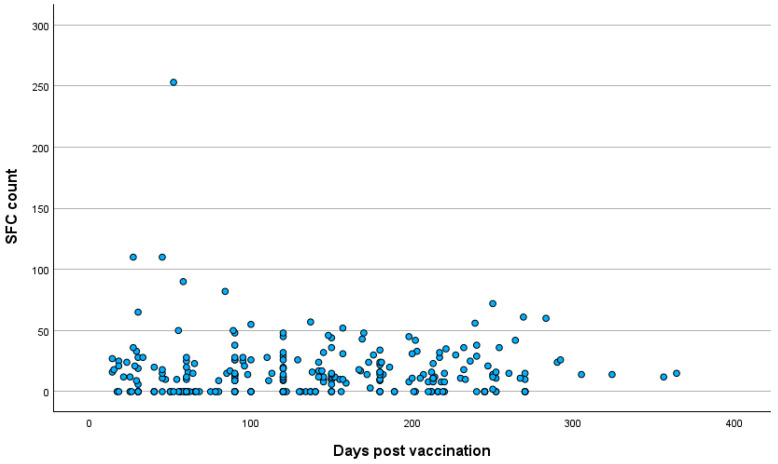
Distribution of T cell response against SARS-CoV-2 S antigen in relation to time period post vaccination in total participants. **Abbreviations:** S antigen—Spike protein antigen (of SARS-CoV-2); SARS-CoV-2—Severe Acute Respiratory Syndrome Coronavirus 2; SFC—spot-forming cell.

**Figure 3:**
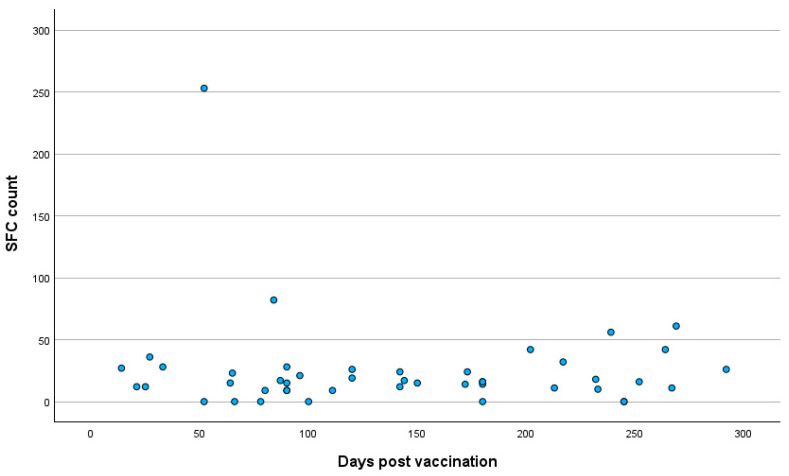
Distribution of T cell response against SARS-CoV-2 S antigen in relation to time period post vaccination in SARS-CoV-2 IgG-positive cases. **Abbreviations:** S antigen—Spike protein antigen (of SARS-CoV-2); IgG—Immunoglobulin G; SARS-CoV-2—Severe Acute Respiratory Syndrome Coronavirus 2; SFC—spot-forming cell.

**Figure 4:**
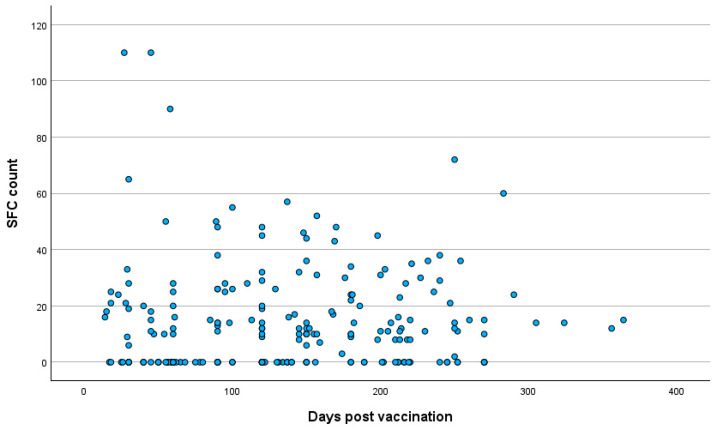
Distribution of T cell response against SARS-CoV-2 S antigen in relation to time period post vaccination in SARS-CoV-2 IgG-negative cases. **Abbreviations:** S antigen—Spike protein antigen (of SARS-CoV-2); IgG—Immunoglobulin G; SARS-CoV-2—Severe Acute Respiratory Syndrome Coronavirus 2; SFC—spot-forming cell.

The mean time period post vaccination for all participants was 137.12 ± 78.7 days (range: 14–364 days). Between groups A and B, no significant difference was observed in the time period post vaccination, with the mean times after vaccination being 136.38 ± 78.68 days in group A and 140.6 ± 79.5 days in group B (T-test, *p* = 0.74). The distributions of the T cell response against the S antigen in relation to the time period post vaccination in the total participants and in the two groups are presented in Figure 2, Figure 3 and Figure 4.

Also, 132/262 (50.3%) exhibited positive cellular and negative humoral reactions, while 7/262 (2.6%) exhibited only a humoral reaction. Additionally, 39/262 (14.8%) tested positive to both responses, and 84/262 (32%) were negative with respect to both reactions (*X*^2^ test, *p* = 0.002).

There was no significant difference in sex proportion between the two groups. In group A, the sex distribution was 118/216 females (54.6%) and 98/216 males (45.4%), while in group B, it was 26/46 females (56.5%) and 20/46 males (43.5%) (*X*^2^ test, *p* = 0.87). Similarly, no significant difference in age was observed between the two groups. The mean age in group A was 58.83 ± 15.63 years, while in group B, it was 62.43 ± 14.67 years (T-test, *p* = 0.15). The study participants’ demographic characteristics and medical information are presented in Table 1.

### 3.2. T Cell Positivity Rate Between Groups

**Figure 5:**
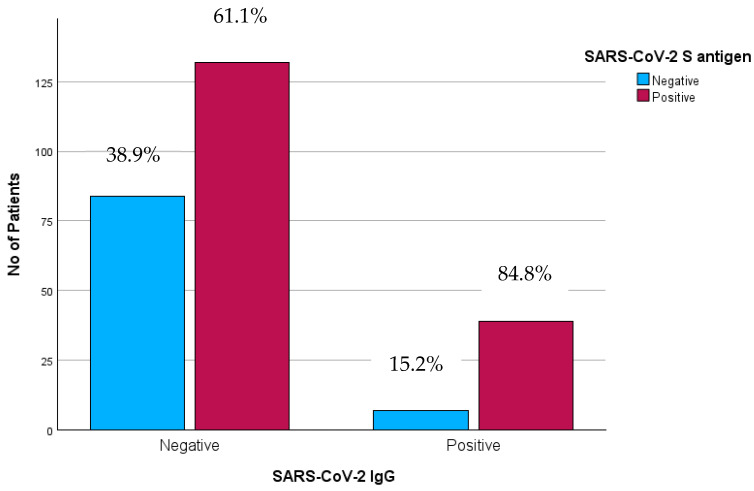
Positivity rate of SARS-CoV-2-specific T cell response among 2 groups. **Abbreviations:** S antigen—Spike Protein Antigen (of SARS-CoV-2); IgG—Immunoglobulin G; SARS-CoV-2—Severe Acute Respiratory Syndrome Coronavirus 2, No—number.

Individuals with a positive IgG antibody response demonstrated a statistically significant higher proportion of positive T-SPOT responses compared to those with a negative antibody response. More specifically, among the two groups, a positive T cell reaction was reported in 132/216 (61.1%) participants in group A and 39/46 (84.8%) in group B (*X*^2^ test, *p* = 0.002) (Figure 5). The T-SPOT results for the N antigen showed a count ≤4 for the total participants.

### 3.3. Quantitative SARS-CoV-2-Specific IFN-g Response

**Figure 6:**
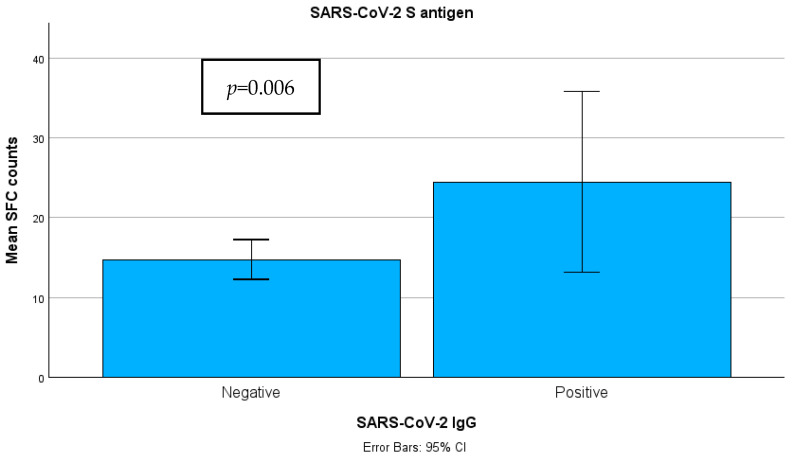
Quantitative SARS-CoV-2-specific T-SPOT results among 2 groups. **Abbreviations:** S antigen—Spike Protein Antigen (of SARS-CoV-2); IgG—Immunoglobulin G; SARS-CoV-2—Severe Acute Respiratory Syndrome Coronavirus 2; T-SPOT—T cell Spot Test; SFC—spot-forming cell.

The median SFC count for the S antigen was 10.00 (n = 216, range 0–110) in group A and 16 (n = 46, range 0–253) in group B (Figure 6). The Mann–Whitney test showed a statistically significant difference in SFC count between the different groups, with a mean SFC count of 125.70 for group A and 158.73 for group B (*p* = 0.006).

### 3.4. Correlation Analysis

**Figure 7:**
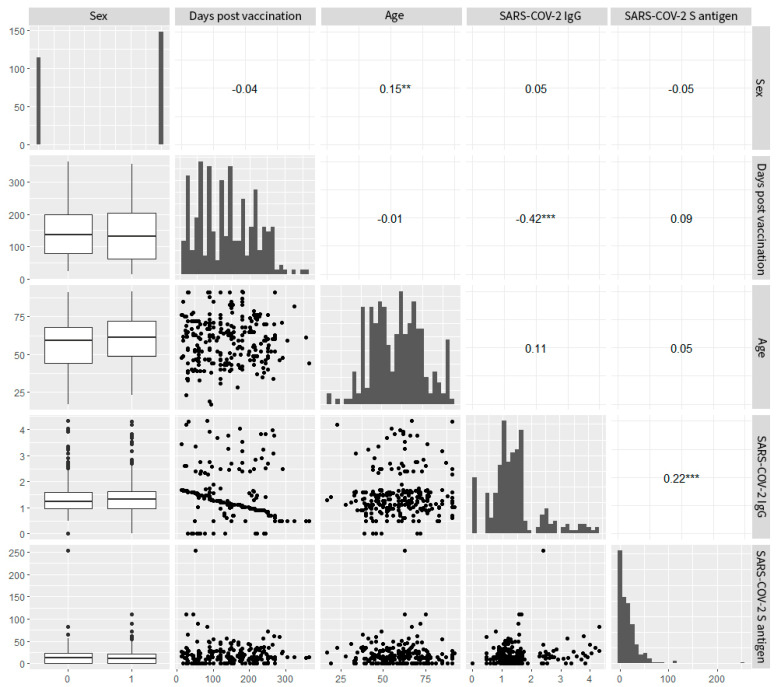
Pair plots. In the diagonal lower half: pairwise scatterplots or whisker plots; on the diagonal: distribution histograms per variable; in the upper half: Spearman’s ρ correlation coefficients for continuous variables or point biserial r coefficient for categorical variables. *** a level of significance < 0.001, ** a level of significance < 0.01. **Abbreviations:** ρ (Spearman ρ)—Spearman’s rank correlation coefficient (used to measure statistical correlation); r (point biserial r)—point biserial correlation coefficient (used for correlation between a binary and continuous variable).

A correlation analysis was conducted to examine potential relationships between variables in the overall and group samples. For the total participants, the variables’ pair plots and their corresponding correlation coefficients (Figure 7) showed a medium strong but strongly significant correlation between IgG levels and the number of days post vaccination (Spearman’s ρ = −0.42, *p* < 0.001). This suggests a time-dependent reduction in IgG levels. The same was not true for cellular immunity levels (Spearman’s ρ = 0.09, *p* > 0.05), suggesting either stability in time or independence from time or both. Nonetheless, a weak but strongly significant correlation between IgG and T cell levels was observed (Spearman’s ρ = 0.22, *p* < 0.001), suggesting a weak positive interaction between the components of humoral and cellular immunity. This positive interaction was more pronounced in group B, where the statistically significant correlation became moderate in strength (Spearman’s ρ = 0.41, *p* < 0.03). Additionally, there was a weak but significant correlation between sex and age (point biserial r = 0.15, *p* < 0.01). Female participants were slightly older on average. No other significant correlations were noted between the remaining variable pairs. The data derived from the analysis of total participants are presented in Figure 7.

### 3.5. Regression Analysis

A logistic regression analysis was conducted to determine the association of age, days post vaccination, IgG antibody titers, sex and comorbidities (e.g., obesity, diabetes, cardiovascular diseases) (see Table 1) with SARS-CoV-2 cellular immunity levels. The dependent variable in the logistic regression model was the presence of detectable SARS-CoV-2 IgG antibodies as a categorical variable. Among the variables examined, cellular immunity levels emerged as the only significant predictor of antibody presence, showing a positive association (B = 0.015; SE = 0.005; Wald= 4.787; *p* = 0.02; Exp(B) = 1.018). Conversely, age, sex, the number of days post vaccination and the key comorbidities of participants did not demonstrate statistically significant associations with a positive IgG antibody response, indicating that these factors alone may not be reliable indicators of humoral immunity in this cohort.

After applying Bonferroni correction for multiple comparisons (adjusted significance threshold: *p* < 0.0056), only the difference in T cell positivity between IgG groups (*p* = 0.002) and the correlations between IgG levels and both time since vaccination (*p* < 0.001) and T cell levels (*p* < 0.001) remained statistically significant.

## 4. Discussion

SARS-CoV-2-specific responses evaluated by the detection of T cell and antibody responses are proven to provide significant protection from SARS-CoV-2 infection. Particularly for vaccinated SARS-CoV-2-uninfected individuals, corresponding comparisons of cellular and humoral responses could be informative for understanding immunity dynamics against COVID-19 [23,24,25,26]. Accordingly, herein, we compared the levels of T cell responses in the presence or absence of humoral immunity in vaccinees naive to SARS-CoV-2. Our results showed that higher SARS-CoV-2 IgG levels correlate significantly with higher levels of SARS-CoV-2-specific cellular responses in fully vaccinated, COVID-19-naive individuals, highlighting a close connection between long-lasting vaccine-induced humoral immunity and robust cellular immunity. Furthermore, while SARS-CoV-2 IgG levels decline significantly with time since vaccination, the same is not true for SARS-CoV-2-specific cellular responses.

These findings imply that cellular immunity persists longer than humoral immunity following vaccination and possibly plays a critical role in sustaining a protective IgG response.

Several studies have demonstrated the protective effects of humoral immunity; however, the levels of neutralizing antibodies decrease 3 to 6 months after the second dose [27,28,29]. Similarly, in vaccinated individuals naive to COVID-19 infection, although a peak initial antibody level was reached, a significant decline each month for the first 6 months was observed, regardless of the vaccination regimen [30]. In another study, humoral responses waned rapidly in vaccinated individuals naive to SARS-CoV-2 infection, while T cell responses were preserved and remained approximately at the same level several months after complete vaccination. As presented therein, 71.8% of the vaccinated participants naive to infection had a positive SARS-CoV-2-specific cellular response 9 months after vaccination. In the same group, individuals with anti-SP IgG antibody levels below 264 BAU/mL had a median of 0.21 IU/mL of IFNγ vs. 0.29 IU/mL in individuals with more than 264 BAU/mL [10]. In an early study for SARS-CoV-2-naive individuals subjected to complete mRNA vaccination, anti-S IgG and antigen-specific CD4+ and CD8+ T cell responses were present up to 6 months post vaccination. Interestingly, peak antibody levels were significantly correlated with long-lasting antibody responses, while long-term humoral immunity was observed upon early CD4+ T cell responses [11]. In a recent study, antibody responses were detectable for up to 7 months, with a gradual decrease 3 months post vaccination and 30% of participants having lost their neutralization activity at 7 months, regardless of the vaccine administered. Importantly, it was also demonstrated that high levels of and long-term antibody responses were associated with the induction of significantly strong antigen-specific CD4+ T cell levels [31]. In another study, T cell levels correlated significantly, but moderately, with anti-SARS-CoV-2 IgG antibodies for a time period up to 9 months post mRNA vaccination [14]. All the abovementioned findings are in line with our results, suggesting that the presence of adequate antibody levels is positively correlated with strong cellular responses, thus highlighting the coordination between both arms of the immune response and the induction of a more robust cellular immunity upon antibody persistence.

There is a general tendency in the vaccine-induced immunity seen in the SARS-CoV-2 infection-naive population wherein neutralizing antibodies wane while T cell responses persist over time after complete vaccination. As demonstrated in a recent study, the levels of spike-specific T cell responses were considerably high 6 months after the second vaccination. In the same time period, the median SARS-CoV-2 spike IgG titer decreased 5.6-fold [32]. Similarly, a significant waning of antibody levels in almost all participants was observed, while stable polyfunctional SARS-CoV-2 spike-reactive T cell responses were present up to 180 days post complete vaccination, irrespective of the regimen [19]. On the contrary, detectable neutralizing antibody responses did not seem to correlate strongly with T cell responses to spike protein in SARS-CoV-2-naive vaccinees approximately 13 weeks post mRNA vaccination [33]. The persistence of T cell responses in individuals with reduced or absent SARS-CoV-2 IgG antibody production may be explained by several immunological mechanisms. Memory T cells can be generated independently of robust antibody production, allowing cellular immunity to persist even when circulating antibodies are undetectable [34]. Some individuals may also exhibit cross-reactive T cell responses due to prior exposure to endemic human coronaviruses, which could contribute to baseline T cell activity [35]. Lastly, factors such as age-related immune changes or subtle impairments in B cell function may result in reduced antibody responses while still permitting an effective T cell-mediated response [36].

The present study bears certain limitations, with the most significant being the lack of additional data regarding the exact type of vaccine the participants had received or other clinical and demographic features; however, it is known that the vast majority were administered with an mRNA-based vaccine. In addition, our findings emerged from a single-center, observational, retrospective study, investigating the dynamics of humoral and cellular immunity during a certain time interval wherein no booster doses were available to study participants. We acknowledge that a retrospective approach inherently limits the ability to establish causal inferences and control over certain variables. Moreover, we acknowledge that using retrospective data can introduce selection bias, particularly as the cohort consists of healthcare-seeking individuals who may differ from the general population in terms of health behaviors and access to care. To mitigate this potential bias, we employed consecutive sampling, which aimed to include all eligible individuals who underwent COVID-19 immunity screening at our healthcare center during the study period. While this approach helps reduce bias, we recognize that selection bias may still influence the results. However, despite these limitations, we believe that our study provides valuable insights into the immune responses in a real-world cohort. Another key limitation of our study is the lack of systematic data on breakthrough SARS-CoV-2 infections, which prevented us from evaluating the direct correlation between immune responses (T cell and antibody) and clinical protection. Future studies incorporating follow-ups on infection outcomes are needed to address this important aspect. We also should acknowledge that conducting multiple comparisons increases the risk of Type I error. While Bonferroni correction was applied to adjust for this, some results that were initially significant did not retain significance under the corrected threshold. This underscores the need for a cautious interpretation of these findings and highlights the importance of further studies to confirm these associations. Lastly, another limitation of this study is the relatively short follow-up period of ≤12 months, which may not fully capture the long-term durability of T cell immunity and the absence of neutralization assays, as IgG levels do not directly correlate with functional neutralization capacity.

## 5. Conclusions

Our findings support a positive correlation between long-lasting vaccine-induced humoral immunity and robust cellular immunity. Furthermore, our study indicates that T cell immunity may persist even when antibody levels are negative, reinforcing the importance of cellular immunity in long-term protection against COVID-19. Characterizing the response of healthy individuals to vaccines is essential to determine future vaccination policies, while the identification of vulnerable non-responders can inform additional interventions, such as extra booster doses of vaccines, while preventing the side effects of immunity overactivation in individuals with robust immunity.

## Figures and Tables

**Table 1:** Descriptive data of participants in study groups.

VARIABLES	Group A (N = 216)	Group B (N = 46)	*p*-Value
Demographic characteristics			
Sex (F/M)	118/98	26/20	NS
Age (years ± SD)	58.8 ± 15.6	62.4 ± 14.6	NS
**Comorbidities**			
Respiratory disorders	32 (14.8)	3 (6.5)	NS
Cardiovascular diseases	30 (13.8)	3 (6.5)	NS
Central nervous system disorders	1 (0.4)	0	NS
Diabetes mellitus	12 (5.5)	3 (6)	NS
Hypertension	28 (12.9)	7 (15.2)	NS
Lipidemia	52 (24)	9 (19.5)	NS
Obesity	27 (12.5)	8 (17.4)	NS

**Abbreviations:** F/M—female/male; NS—Not Significant; SD—Standard Deviation; N—number.

## Data Availability

All data from this study are included in this article.
